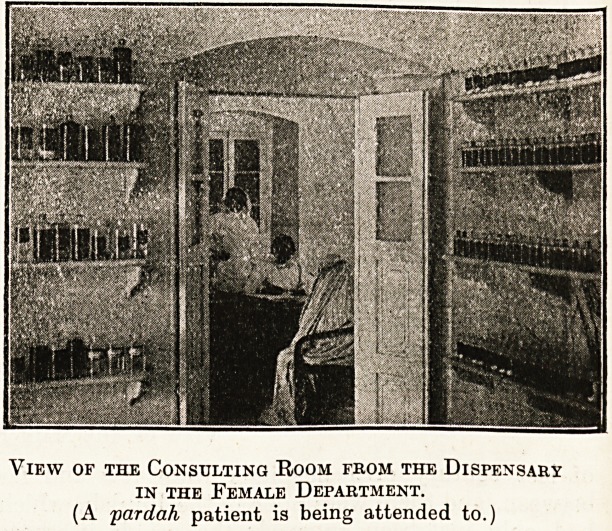# Building a Hospital in India

**Published:** 1915-04-17

**Authors:** C. H. Buck

**Affiliations:** I.A., Deputy Commissioner.


### BUILDING A HOSPITAL IN INDIA.

### I.—The Story of the Karnal District Hospital.

By Major C. H. BUCK, I.A., Deputy Commissioner.

India offers a wide field for the exercise of hobbies, and an executive member of the Indian Civil Service who happens to be fond of engineering can find plenty of scope for putting into practice such knowledge of this science as he may have gained; for, as president of a District Board and controller of municipalities, the civilian has to arrange for the construction of a large variety of public works.

I have been responsible for the construction of many buildings, but none gave me more interest than the large hospital at the headquarters of the Karnal District in the Punjab, which was completed in 1913 as a memorial to his late Majesty, King Edward VII.

### Growth of Hospital Provision.

Medical work in India has made extraordinary progress during the past fifty years. In the middle of last century civil hospitals were few and far between, and there were none in the Punjab, which had only just been annexed; the people in those days had to depend on the village barbers for rough surgical operations, and on native physicians, who were often illiterate, for other medical treatment; now each district in India has a hospital at its headquarters, and dispensaries, with some accommodation for in-patients, in the outlying tracts, while the principal cities are well provided.

Excluding military and railway institutions and those maintained by Government for the police and other departments, there are at the present time nearly 3,500 hospitals and dispensaries open to the public; these, however, are not maintained by charity, as in England, but chiefly by local funds. More than 600,000 indoor and actually over 30,000,000 outdoor patients are treated in these institutions every year. Travelling dispensaries are also being introduced to tour and provide treatment for the people in unhealthy tracts and in places where epidemics may occur. There is, however, a vast amount yet to be done, and a huge sum of money will be required before medical relief can be brought within reach of each of the three hundred million inhabitants of India.

The Karnal District, which is situate just north of the new province of Delhi, has a population of about 800,000, and for a long time the want of a large hospital at its headquarters had been felt; formerly it could only boast of a few dilapidated buildings, crowded together in a confined space near the town, which were most unsuitable for the proper treatment of patients.

For several years funds were collected for the construction of a new hospital, and finally, in 1910, it was resolved to erect one as a memorial to King Edward. A sum of two and a half lakhs of rupees, or nearly £17,000, was scraped together, of which one and a half lakhs were contributed by the District Board and over half a lakh subscribed by the public; two local magnates presented a fine site of nineteen acres, and we were ready to start work early in 1911.

View of the Main Block and One Ward.

### Indian Peculiarities.

Before venturing to design the buildings I had not only visited several English hospitals, but had noticed the good and bad points of a large number of similar institutions in India and had discussed their peculiarities with numerous civil surgeons. It was also necessary to consult Indians about their customs, in order to make everything suitable for use by them, as they do many things in exactly the opposite way to English people.

For instance, the Indian turns a wheel and similar articles in the contrary direction to the movement of the hands of a clock; he drives a drill in this way and his saws are made to pull instead of to push, while he prefers a key which turns in the opposite way to ours; he performs all culinary operations while squatting on the floor, and Indian workmen generally adopt this position. Thus the ranges in the kitchens have to be on the floor level

Some of the Staff.
(Dr. Charters, civil surgeon, with the assistant surgeon and sub-assistant, and the female sub-assistant surgeon and her compounder.)

and to be made in native fashion; the stone slabs at the *dhobi's ghat*, or washerman's platform, have to be so arranged that the clothes can be swung on to them over the left shoulder. Windows with sashes are unknown in India, and all windows and doors are made in two flaps to open inwards; those which are not sheltered by verandahs are provided with shades to keep out rain and the direct rays of the sun. The floors of a hospital must slope properly to allow flushing, and, of course, all corners have to be rounded; there must be no superfluous ledges where dust can lodge; perforated zinc and wire gauze are necessary to keep out flies and other insects, particularly from the wards and from places where food is kept. The shop in the hospital enclosure is practically surrounded with wire gauze, for it is from here that most of the food supplies for the staff and patients are obtained. As for the drains, they are all open, for these are preferable in India when there is not a sufficient flush available; solid refuse is removed in hermetically closed iron buckets on trolleys or carts and trenched in the fields.

### Home-made Building on the Spot.

All of these matters and innumerable others had to be considered in preparing the designs. The chief difficulties, however, arose when the actual building operations commenced, for every portion of the work from the laying out and digging of the trenches for the foundations to the finishing touches of "Paripan" in the interior had to be scrutinised most carefully; in fact one frequently had to learn how to do a thing oneself and then instruct the artificers.

The contractor engaged a small army of brick-makers, masons, carpenters, blacksmiths, and workmen, skilled and unskilled, of all descriptions; practically everything, with the exception of the steel beams for the roofs, the waterworks, fittings, glass, and furniture, was made on the spot. The bricks and lime were burnt in kilns close to the site, and for a period of two years the immediate neighbourhood resembled a large ant-hill. The duty of supervision was shared between the district engineer and myself, and one or other of us inspected the work almost daily in our spare moments. On several occasions portions of the work, which were hurriedly run up during our absence on tour, had to be demolished and rebuilt owing to defects which were discovered on our return. The ordinary Indian contractor in Northern India has certainly not yet discovered that it pays to supply good material and do sound work.

In that part of the world one has also to contend with a variety of pests which are unknown in Europe, but far and away the worst, from the house-owner's point of view, is the white ant; its ravages are awful; it eats and destroys almost everything which is not mineral, leaving little streaks or tubes of earth instead of the original substance.

Brilliant sunshine, with dry and intense heat for several months in the summer, then continual and heavy rain during the monsoon, followed by dry

View of the Consulting Room from the Dispensary in the Female Department.
(A *pardah* patient is being attended to.)

cold in the winter; all these had to be considered when designing the buildings.

Thick walls and broad verandahs, a high plinth and jack-arched flat roofs, covered with concrete, cement, and a thick layer of earth mixed with crushed straw, are perhaps as good as anything for dealing with the variations of temperature and climate. Against the white ants and damp, a course of pitch was placed in the walls just above the foundations; the slabs of the floors were laid on concrete, well tarred, and below this was plenty of sand; but, notwithstanding these precautions, the persistent little creatures forced their way, even between marble slabs, into some of the rooms in their efforts to reach the woodwork, of which they are remarkably fond.

(*To be continued.*)

## Figures and Tables

**Figure f1:**
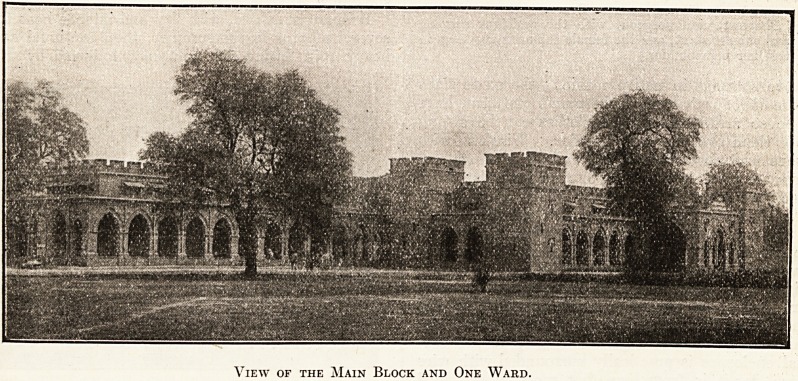


**Figure f2:**
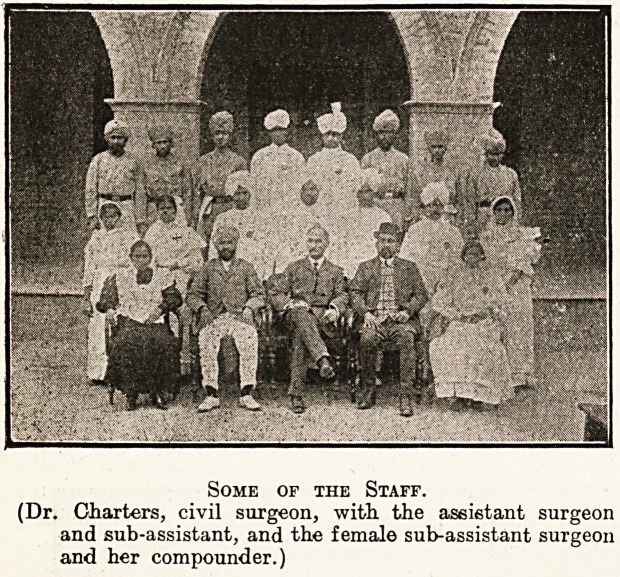


**Figure f3:**